# A comprehensive overview of minimal residual disease in the management of early-stage and locally advanced non-small cell lung cancer

**DOI:** 10.1038/s41698-025-00984-9

**Published:** 2025-06-13

**Authors:** Aristeidis E. Boukouris, Kleita Michaelidou, Simon A. Joosse, Andriani Charpidou, Dimitrios Mavroudis, Konstantinos N. Syrigos, Sofia Agelaki

**Affiliations:** 1 Department of Medical Oncology, University General Hospital of Heraklion, Vassilika Vouton, Crete Greece; 2 Laboratory of Translational Oncology, School of Medicine, University of Crete, Vassilika Vouton, Crete Greece; 3 Department of Tumor Biology, University Medical Center Hamburg-Eppendorf, Hamburg, Germany; 4 Oncology Unit, 3rd Department of Internal Medicine, National and Kapodistrian University of Athens, Athens, Greece

**Keywords:** Cancer, Non-small-cell lung cancer

## Abstract

Novel therapies have significantly improved survival for non-metastatic non-small cell lung cancer (NSCLC), however recurrence remains a challenge. Current treatment and surveillance strategies rely on imaging and clinical assessments with limited sensitivity in early detection of disease progression. Liquid biopsy-mediated detection of minimal residual disease (MRD) allows monitoring of tumor activity at the molecular level before clinical and radiologic progression. Here, we review the current evidence for MRD in the adaptive management and surveillance of non-metastatic NSCLC, focusing on the missing links that prevent its widespread clinical adoption.

## Introduction

Lung cancer ranks first among other cancer types in terms of incidence and mortality, according to the latest global cancer statistics^1^, with almost 85% of all cases representing non-small cell lung cancer (NSCLC). The estimated 5-year survival rate for all NSCLC stages combined is approximately 25%, primarily due to the low survival rates of inoperable stage III and stage IV disease (20 and 6%, respectively), which account for more than half of new NSCLC cases. By contrast, in early-stage NSCLC, defined as localized or locally advanced resectable disease, survival rates can be as high as 75% in younger patients with stage I disease^2^. However, in the event of relapse, which affects between 20 and 50% of stage I-IIIA patients^3,4^, 5-year survival rates do not exceed 30%^5–7^. Therefore, both prevention and early detection of recurrence remain critical in the management of early-stage NSCLC. These principles also apply to locally advanced, unresectable disease, where treatment strategies increasingly focus on intensifying systemic therapy and optimizing surveillance.

Recent advances in the treatment of early-stage NSCLC reflect the concerted effort to minimize the risk of recurrence and to improve long-term survival. The introduction of neoadjuvant and perioperative immunotherapy-based strategies was driven by the hypothesis that the presence of the primary tumor and an intact lymphatic system prior to surgical resection can stimulate a more robust and durable immune response, ultimately translating into reduced recurrence rates and improved survival. Indeed, these approaches have significantly improved RFS across different regimens^8^. In oncogene-driven NSCLC, including tumors harboring *EGFR* or *ALK* alterations, adjuvant tyrosine kinase inhibitors (TKIs) have also achieved a significant reduction in the risk of recurrence^9,10^, while neoadjuvant TKI-based regimens are currently undergoing clinical testing^11^. Additionally, questions remain regarding the optimal duration of adjuvant TKIs^12,13^ and immunotherapy^14,15^, particularly in relation to the degree of achieved pathologic response. Currently, standard-of-care post-treatment surveillance includes routine imaging and clinical evaluations at 6-month intervals, aiming at detecting disease recurrence at an early stage^16,17^.

The major limitation of existing follow-up strategies is that they can only inform treatment decisions based on the presence of radiologically and/or clinically measurable disease, which requires the presence of a substantial amount (millions) of cancer cells, i.e. increased tumor burden^18^. This also increases the possibility of genetic diversity as more cancer cells with clonal heterogeneity accumulate over time. In fact, the landmark TRACERx study, which evaluated patterns of early metastatic dissemination in NSCLC, revealed through simulation models that early metastatic clonal divergence can occur even in small tumors measuring less than 8 mm^19^. Therefore, due to clonal heterogeneity, the efficacy of therapeutic interventions can be substantially compromised when solely based on radiologic recurrence.

Τhe necessity to lower the detection threshold of residual disease before radiographic and clinical progression occurs, can be addressed by liquid biopsy. Liquid biopsy is a minimally invasive procedure aimed at detecting circulating tumor cells (CTCs) or tumor-related material (e.g., circulating tumor DNA, ctDNA; cell-free tumor RNA, ctRNA; extracellular vesicles, EVs and metabolites) using a variety of molecular techniques^20^. As a result, the term minimal residual disease is often used interchangeably with molecular residual disease. Recently, liquid biopsy techniques have rapidly evolved and achieved increased sensitivity of MRD detection in lung cancer, primarily *via* quantification of ctDNA^21^. Efforts using CTCs in NSCLC have lost ground compared to ctDNA, mainly due to the rarity and heterogeneity of CTCs, which pose significant challenges regarding their detection and accurate characterization.

In parallel, mounting evidence suggests that MRD status correlates with survival outcomes in NSCLC, leading to its incorporation in randomized clinical trials (RCTs) as a stratification and predictive biomarker^22^. However, despite progress in optimizing MRD detection and understanding its potential use for informed clinical decision-making in NSCLC, it has not as yet been adopted in official guidelines. Important questions remain regarding the optimal introduction of liquid biopsy and MRD detection in adaptive surveillance and treatment personalization for early-stage NSCLC.

**Fig. 1:**
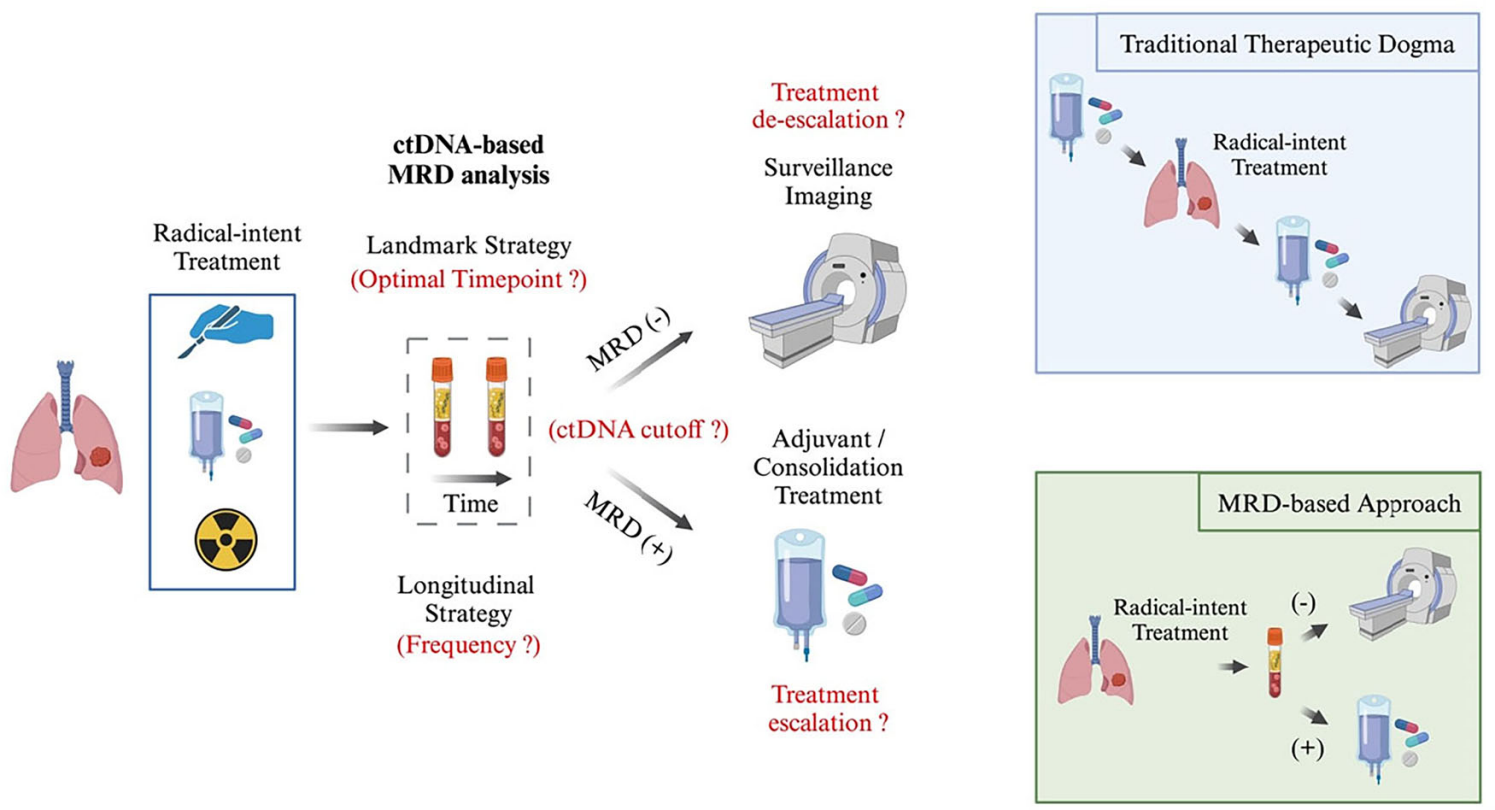
Hypothetical schematic workflow for MRD-guided management of early-stage (or locally-advanced, inoperable) NSCLC in the near future. Question marks denote areas of uncertainty that warrant further investigation. Insets (right side) illustrate the gross differences between the existing therapeutic approach and a potential future MRD-based strategy. Created in BioRender. Boukouris, A. (2025) https://BioRender.com/1fk6hfs.

This review focuses on the current evidence supporting the use of MRD in the management of non-metastatic NSCLC and outlines the missing steps required for its clinical adoption [Fig. 1]. The majority of the data presented herein concern early-stage NSCLC, as previously defined. However, where necessary, reference will also be made to the few existing MRD studies conducted in locally advanced, inoperable disease, a disease stage which can still be managed with curative intent in some patients. Before delving into these clinical data, a smaller section is dedicated to the principles of ctDNA-based MRD detection technology that is currently utilized in NSCLC and other malignancies.

## Detection of MRD using ctDNA analysis

The concept of MRD was first described in hematological malignancies more than 40 years ago. Since then, technological advancements in the field of MRD detection have expanded its applicability beyond hematological cancers to a wide range of solid tumors, including lung cancer^23^. To date, numerous MRD detection assays and tests have been developed, which vary in sensitivity, specificity, turn-around time, susceptibility to technical biases, and the degree of standardization across laboratories [Table 1].

**Table 1:** Commercially available MRD detection assays

Detection method	Tumor-informed or -naive	Methodological principle	Sensitivity (detection limit)	Selected references
Droplet Digital PCR (ddPCR)	Tumor-informed	Allele-specific PCR in thousands of microdroplets for precise detection of known mutations	~0.01% variant allele frequency (VAF) detectable ( ≈ 1 mutant in 10^4 wild-type copies); in optimized setups, as low as ~0.001% VAF	PMID: 37686024 PMID: 34985936
Signatera (Natera)	Tumor-informed	Whole-exome sequencing (WES) of tumor and normal tissue to identify patient-specific mutations, followed by a multiplex PCR-based custom panel (16 clonal tumor variants) tracked in plasma via deep sequencing	~0.01% VAF	PMID: 38824449
RaDaR (Inivata/NeoGenomics)	Tumor-informed	Whole-genome sequencing of tumor to select up to 48 tumor-specific mutations, followed by ultra-deep sequencing (amplicon-based) of those variants in plasma	~0.001% VAF	PMID: 37052271 PMID: 37686024
PhasED-Seq (Foresight Diagnostics)	Tumor-informed	Tracks phased variants – multiple mutations on the same DNA fragment. By requiring concordant detection of multiple SNVs in one fragment, it achieves error profiles ~100× lower than single-SNV methods	~0.0001% VAF	PMID: 37056631 PMID: 34294911
MRDetect (C2i Genomics)	Tumor-informed	Low-depth whole-genome sequencing (WGS) of tumor and plasma with AI-driven analysis to detect subtle tumor-specific genomic patterns (SNVs/CNAs) in plasma	~0.001% VAF	PMID: 37056631
FoundationOne® Tracker (Foundation Medicine)	Tumor-informed	Personalized ctDNA test using whole-genome/exome sequencing of tumor to select specific mutations for longitudinal plasma tracking	~0.01% VAF	PMID: 38824449
TARDIS (Tumor-Aware ctDNA Detection for Improved Survival)	Tumor-informed	Targeted digital sequencing of 8–16 known tumor mutations with ultra-deep sequencing	~0.002% VAF	PMID: 31391323
CAPP-Seq (Cancer Personalized Profiling by Sequencing)	Tumor-informed or Tumor-naïve	Hybrid-capture NGS targeting common cancer mutations (~100–200 genes in NSCLC); deep sequencing with error suppression	~0.003% VAF	PMID: 37686024
Guardant Reveal™ (Guardant Health)	Tumor-naïve	“Plasma-only” ctDNA assay combining targeted sequencing of a fixed ~500 kb panel of cancer genes with detection of abnormal methylation patterns	~0.01% VAF	PMID: 37052271
PGDx elio™ Plasma Complete (Personal Genome Diagnostics/Labcorp)	Tumor-naïve	Hybrid-capture NGS assay profiling 521 cancer-related genes in plasma, assessing SNVs, CNAs, and fusions	~0.1% VAF	PMID: 37939112
Roche AVENIO (Surveillance Panel)	Tumor-naïve	Fixed-panel hybrid-capture NGS assay (~197 genes) optimized for broad tumor profiling and MRD detection	~0.1% VAF	PMID: 37939112
DELFI (DNA Evaluation of Fragmentation)	Tumor-naïve	Genome-wide cfDNA fragmentation profiling using machine learning	Sensitivity ~70–90% (not VAF-based)	PMID: 31142840

This table presents a comparative overview of commercially available MRD detection assays, distinguishing between tumor-informed and tumor-naïve approaches. Tumor-informed assays generally achieve higher sensitivity by tracking patient-specific tumor mutations, whereas tumor-naïve methods offer broader applicability without requiring tumor tissue sequencing.

*AI* artificial intelligence, *cfDNA* cell-free DNA, *CNA* copy number altered, *ctDNA* circulating tumor DNA, *MRD* minimal residual disease, *NGS* next-generation sequencing, *NSCLC* non-small cell lung cancer, *PCR* polymerase chain reaction, *SNV* single nucleotide variant.

### ctDNA analysis for MRD evaluation in solid tumors

For solid tumors, MRD detection assays primarily rely on the analysis of ctDNA, a tumor-derived subset of cell-free DNA (cfDNA) and a key analyte in liquid biopsy^24–26^. Detection of ctDNA in blood is particularly challenging, as it constitutes a small fraction of total cfDNA. In fact, ctDNA levels range from ≥5 to 10% of cfDNA in late-stage cancers to as low as ≤0.01–0.1% in early-stage cancers or early post-surgical recurrence^27^. Due to these challenges, the U.S. Food and Drug Administration (FDA) has approved ctDNA-based comprehensive genomic profiling of NSCLC only when tumor tissue is unavailable or at the time of disease progression.

Given its rarity, ctDNA-based MRD tests are designed to employ highly sensitive technologies such as droplet digital PCR (ddPCR) and next-generation sequencing (NGS), capable of detecting even minimal traces of ctDNA among the abundant background of non-tumor-derived cfDNA^26^. ddPCR offers higher sensitivity, with the ability for absolute quantification of target DNA and the detection of mutant allele frequencies (MAF) as low as 0.001%. However, it is restricted to detecting only a predefined set of mutations, limiting its applicability in broader genomic assessments. On the other hand, NGS provides extensive genomic coverage, enabling the simultaneous detection of both known and novel mutations. Although its sensitivity is lower than ddPCR, newer methods, such as hybridization capture-based approaches (e.g., CAPP-Seq, Cancer Personalized Profiling by Deep Sequencing) and PCR amplicon-based NGS (e.g., Safe-Seq, Safe Sequencing System) have significantly improved detection capabilities, achieving sensitivity limits as low as 0.02% MAF^25,26,28,29^.

### MRD assessment via ctDNA: tumor-informed and tumor-naïve approaches

There are two main approaches for ctDNA-based MRD evaluation, tumor-informed and tumor-naïve (or tumor-agnostic), which differ in their reliance on prior tumor sequencing. The choice between these strategies depends on various factors, including study objectives, tumor tissue availability, required sensitivity, and cost considerations^30^.

#### Tumor-informed approaches and related platforms

Tumor-informed methods rely on patient-specific genomic profiling of tumor tissue, using techniques such as whole-genome sequencing (WGS), whole-exome sequencing (WES), or large NGS panels. These methods identify tumor-specific mutations that are then tracked longitudinally in plasma using bespoke assays, such as custom PCR panels or targeted NGS. This approach offers high specificity, minimizing false positives from non-tumor mutations like those arising from clonal hematopoiesis of indeterminate potential (CHIP). However, tumor-informed methods require sufficient high-quality tumor tissue, involve longer assay development times, and may not capture newly emerging, therapy-relevant mutations arising from tumor heterogeneity or clonal evolution^25,31,32^.

Key platforms that exploit tumor-informed approaches for MRD detection include Signatera™ (Natera), RaDaR™ (Inivata/NeoGenomics), and ArcherDX PCM (Invitae), all of which employ amplicon-based targeted NGS with a limit of detection (LoD) as low as 0.001–0.02%^31,33^. Despite their advantages, WES-based platforms can exhibit uneven coverage across challenging genomic regions, leading to the potential omission of clinically significant variants. To overcome these limitations, WGS-based tumor-informed platforms such as MRDetect™ (Veracyte), C2-Intelligence™ (C2i Genomics), and NeXT Personal (Personalis) offer broader genomic coverage (>1000 targetable variants in plasma) and leverage advanced computational methods (e.g., AI-based algorithms) to enhance sensitivity (LoD as low as 0.0001% tumor fraction)^34,35^. Finally, hybrid capture-based platforms like PhasED-Seq™ (Foresight Diagnostics) and MAESTRO (Adela Bio) utilize phased variants to achieve sensitivity below 0.0001% tumor fraction^25,36^.

#### Tumor-naïve approaches and related platforms

Tumor-naïve (agnostic) methods are blood-based assays that do not require prior tumor sequencing. Instead, they use predefined panels of recurrent cancer-associated genomic or epigenomic alterations, such as common driver mutations or DNA methylation patterns^37,38^. These universal panels make tumor-naïve platforms broadly applicable, offering faster turnaround times and lower costs. However, their lack of individualization may reduce sensitivity as patient-specific mutations, unique to heterogeneous tumors, may be missed. Additionally, their broader genomic coverage can increase background noise, necessitating advanced bioinformatic tools, such as unique molecular identifiers (UMIs) and methylation profiling, to enhance specificity and accuracy^36,39^.

Tumor-naïve platforms employ either amplicon-based or hybrid capture-based methods. Amplicon-based platforms, such as InVisionFirst®-Lung (Inivata), SafeSeqS (Sysmex Plasma-Safe-SeqSensei), SiMSen-Seq and the Oncomine™ cfDNA Assay (Thermo Fisher Scientific), provide comparable sensitivity for various applications, achieving a LoD of 0.07–0.33% MAF^26,36^. Hybrid capture-based platforms offer broader genomic perspectives, enabling the analysis of numerous genomic regions simultaneously. A prominent example is Guardant Reveal™ (Guardant Health), a tumor-naïve ctDNA assay designed for MRD detection and recurrence monitoring, with demonstrated clinical validity in colorectal cancer (CRC) and ongoing studies in other solid tumors. The assay integrates genomic and epigenomic alterations to enhance sensitivity, with an approximate LoD of ~0.01% VAF. Similarly, the FDA-approved FoundationOne Liquid CDx™ (Foundation Medicine) achieves a LoD of 0.37–0.9% MAF, and has applications spanning NSCLC, CRC and breast cancer. AVENIO™ ctDNA Assay (Roche Diagnostics), utilizes CAPP-Seq technology with a 197-gene panel optimized for NSCLC and CRC, achieving a LoD of 0.5–1% MAF^25^.

Emerging tumor-naïve technologies, such as Delfi-TF and eTam-Seq™, integrate advanced methodologies, including fragmentomics and methylation profiling, to further enhance sensitivity and specificity. GRAIL Galleri® and OverC® (Burning Rock Dx) extend tumor-naïve applications to early cancer detection, focusing on methylation profiling to classify ctDNA sources and monitor recurrence. Both platforms demonstrate promising utility in MRD and recurrence detection, complementing tumor-informed strategies^36^.

## Clinical utility of MRD in NSCLC: current proof and missing evidence

### Can ctDNA analysis detect MRD following curative-intent treatment in NSCLC?

Several studies have shown that detection of post-treatment MRD in NSCLC using ctDNA analysis is feasible. However, reported performance rates vary substantially across studies depending on a combination of biological characteristics of the disease (stage, histologic subtype), technical parameters (assay type, platform), and sampling time after surgery. Overall, the amount of ctDNA released in the circulation is highly correlated with the tumor burden so that the probability of detection decreases significantly in earlier stages, especially after removal of the tumor bulk^40^. Lower ctDNA shedding has also been associated with the adenocarcinoma histological subtype (*vs*. squamous cell carcinomas)^41^ and the absence of necrosis^42^. In a systematic review of 13 studies that performed ctDNA analysis for post-operative MRD detection in stage I-III NSCLC patients, detection rates ranged between 6 and 46%, with stage I, II and III patients representing approximately 42%, 28 and 30%, respectively of the analyzed subjects^43^. Higher overall sensitivity was achieved in studies that performed longitudinal MRD analysis (serial measurements at multiple time points) compared to landmark (single designated time point) analysis. Interestingly, analysis (tracking) of multiple mutations per patient (identified pre-treatment) can significantly increase sensitivity of post-treatment MRD detection (94% *vs*. 58% for single mutation, *p* = 0.001), as shown by Chaudhuri et al., who utilized a CAPP-seq platform^44^. Of note, the remarkably high detection rates achieved in this study even with single gene (mutation) analysis (58%), were probably also influenced by the fact that 80% of all patients had stage II and III disease.

The suboptimal sensitivity of current MRD assays, leading to a high rate of false-negative results, particularly in stage I disease, remains one of the key barriers to routine clinical implementation. Different assays and strategies to overcome decreased sensitivity are continuously being explored. False positives, which may arise due to CHIP in deep sequencing approaches, should also be considered^40^. Further studies are needed to address which is the optimal MRD detection technology to be utilized in the clinic, which is the optimal sampling time point and to accurately define the ctDNA level cutoffs for reporting residual disease.

### What is the prognostic role of MRD detection following therapy with curative intent?

Mounting evidence from the last few years of research, mainly with ctDNA and to a lesser extent with CTCs, suggests that MRD could serve as a surrogate marker of disease activity and prognostic indicator of survival in resectable NSCLC, with varying accuracy, across different disease stages and molecular backgrounds. Meta-analyses of these data indicate that patients with detectable ctDNA have an increased risk of recurrence and death, especially when ctDNA levels persist in the adjuvant setting after bulk tumor resection^43,45^. Inversely, clearance of ctDNA after radical therapy is associated with prolonged survival^41,45–47^. Obviously, the level of confidence in detecting ctDNA clearance depends on the LoD of the analytical assay used.

Thus, in a large prospective observational study by Zhang et al. (median follow-up period: 19.7 months), an intriguing correlation between MRD and clinical outcome (disease-free survival, DFS) was demonstrated for patients with resectable stage I-IIIA NSCLC (stage I: 62.4%, stage II: 20.3%, stage III: 17.2%). In the landmark time point analysis performed at 1 month (±7 days) after surgery for those who did not receive adjuvant therapy, and at 1 month (±7 days) after the last cycle of chemotherapy for those who received adjuvant chemotherapy, 86.6% of patients with negative MRD status remained disease-free (negative predictive value, NPV: 86.6%). Inversely, 17 out of 21 patients with detectable MRD suffered disease recurrence (positive predictive value, PPV: 81%). When integrating longitudinal time points, the NPV and PPV further increased to 96.8% and 89.1%, respectively^48^.

These associations have been corroborated in several phase II and III RCTs of perioperative immunotherapy and TKIs in the early-disease setting. The IMpower010 study of adjuvant Atezolizumab after adjuvant chemotherapy in resected stage IB-IIIA NSCLC, first showed that ctDNA positivity after surgery and before the administration of adjuvant therapy was a poor prognostic factor for DFS across treatment arms (Atezolizumab, placebo) and PD-L1 levels^49^. Similar results were obtained in the ADAURA study, a large, randomized trial of Osimertinib *vs*. placebo in early-stage *EGFR*-mutant NSCLC after complete surgical resection, which included a pre-planned longitudinal ctDNA analysis via personalized MRD panels (RaDaR, NeoGenomics). MRD detection during Osimertinib treatment had a clinical sensitivity and specificity of 65 and 95%, respectively in predicting DFS^50^.

Notably, an important consideration for ctDNA MRD assays in early-stage NSCLC is the suboptimal NPV for recurrence as compared to their high PPV. Consequently, a negative ctDNA result does not ensure cure of disease. For example, in the IMpower010 trial, during the first 2 years of follow-up, approximately one third of recurrences on either arm occurred in patients with negative ctDNA levels after surgery and chemotherapy^51^. Newer more sensitive assays, such as NeXT Personal with LoD as low as 0.0001–0.0003% tumor fraction^52^ could potentially improve the NPV, however this needs to be further tested in the clinic. Furthermore, the optimal time point for post-operative MRD detection to enable clinical decision-making has not been established in NSCLC. Importantly also, the level of ctDNA that may signify tumor progression has not yet been determined, and needs to be addressed in prospective multicenter studies. It has been suggested that longitudinal ctDNA levels should be dynamically monitored preoperatively, postoperatively and after adjuvant therapy to improve sensitivity and specificity^33^.

The prognostic value of MRD probably extends to locally advanced, inoperable disease, although data are far more immature compared to resectable NSCLC. Jun et al. analyzed ctDNA levels both after chemoradiotherapy and during consolidation immunotherapy in 38 patients with unresectable stage II or III disease that participated in the Big Ten Cancer Research Consortium LUN 16-081 trial. Detection of ctDNA after chemoradiotherapy predicted significantly inferior 2-year PFS (29% *vs*. 65%, *p* = 0.0048), while ctDNA clearance after 1 cycle of immunotherapy correlated with significantly better 2-year PFS, compared to persistent ctDNA positivity (72% *vs*. 0%, *p* < 0.0001)^53^. ctDNA-based exploratory analyses from the ongoing LAURA trial of consolidation Osimertinib following radical chemoradiotherapy for unresectable stage III *EGFR*-mutant NSCLC are awaited^54^.

### What is the prognostic role of ctDNA dynamics in the neoadjuvant setting?

Neoadjuvant chemoimmunotherapy has emerged as an important treatment strategy in resectable NSCLC. Correlative ctDNA analyses of recent practice-changing clinical trials demonstrated the prognostic impact of ctDNA dynamics in the neoadjuvant setting. Thus, in the phase II NADIM study that included patients with resectable stage IIIA disease, clearance of ctDNA after 3 cycles of neoadjuvant Nivolumab plus chemotherapy was significantly correlated with longer progression-free survival (PFS) (HR: 0.16, 95% CI: 0.03–0.73) and overall survival (OS) (HR: 0.05, 95% CI: 0–0.62) (analysis excluding patients with undetectable ctDNA at baseline)^55^. Similar results were reported in the phase III AEGEAN study of perioperative Durvalumab plus neoadjuvant chemotherapy in patients with resectable stage II-IIIB NSCLC, which showed that patients achieving ctDNA clearance after 3 cycles of neoadjuvant therapy had significantly better event-free survival (EFS) outcomes, compared with those with residual ctDNA, especially in the Durvalumab plus chemotherapy arm (HR: 0.26; 95% CI: 0.13–0.54). Intriguingly, additional sets of data from the same study implied for the first time that ctDNA clearance may hold the potential for prognosticating survival regardless of pathologic response, a well-known potential early predictor of survival in NSCLC^56^. More specifically, patients without detectable ctDNA before surgery had significantly better survival outcomes, even in the absence of pathologic complete response (pCR) (Durvalumab arm; ctDNA clearance(+), pCR(+); HR: 0.14, 95% CI: 0.04–0.48 vs. ctDNA clearance(+), pCR(–); HR: 0.35, 95% CI: 0.16–0.76)^57^. However, the potential independent negative prognostic value of ctDNA clearance still requires extensive validation, also considering recent analyses from the phase III CheckMate 816 (neoadjuvant Nivolumab plus chemotherapy) and CheckMate 77T (perioperative Nivolumab plus chemotherapy) trials in resectable NSCLC. In both studies, although clearance of ctDNA was correlated with a higher pCR rate (i.e. in CheckMate 816, pCR was 46% *vs*. 0% in patients with and without ctDNA clearance, respectively), as many as 50% of patients with ctDNA clearance did not achieve pCR, implying that ctDNA negativity may not necessarily translate into eradication of residual tumor cells^58,59^.

Taken together, these results suggest that, while ctDNA clearance is a promising biomarker, it may not reliably reflect complete tumor eradication and thus, as yet, it cannot be used as a definitive indicator of treatment efficacy. Furthermore, further investigation with stage-stratified analyses is required to clarify whether the prognostic significance of ctDNA detection and clearance varies according to disease stage, i.e. between IB-II and later (III) disease stages. Finally, prospective studies are also needed to establish solid criteria for assessing ctDNA dynamics and their correlation with clinically significant endpoints i.e. response to treatment, disease recurrence and survival.

### What is the role of longitudinal MRD surveillance following treatment with curative intent?

Monitoring patients using ctDNA analysis following curative treatment could serve as a minimally invasive surveillance method to track cancer activity and detect molecular recurrence prior to radiologic recurrence, potentially allowing for earlier and more effective therapeutic interventions. To this end, longitudinal ctDNA monitoring has expectedly prevailed over landmark analysis mainly due to: (a) the suboptimal sensitivity of MRD detection (especially for low and/or slow shedders)^40,60^, and (b) the often unpredictable temporal recurrence pattern of early-stage NSCLC, with early relapses mostly occurring during the first 2 years after surgery, while late recurrences (more than 5 years after surgery) are also encountered affecting at least 8–10% of all patients in various cohorts^7,61^.

In early-stage NSCLC, application of longitudinal MRD surveillance has displayed varying degrees of sensitivity in predicting recurrence. In a meta-analysis of 11 studies by Zhong et al., longitudinal ctDNA surveillance in patients with (mostly) resectable stage I-IIIB disease after curative-intent treatment, predicted relapse with a pooled sensitivity of 76% (95% CI: 68–82%) for hybrid capture-based NGS and 77% (95% CI: 64–87%) for amplicon-based NGS^62^. Individual sensitivities ranged from 50 to 100%, probably due to inter-study heterogeneity in terms of disease stage, molecular background, administered (neo)adjuvant therapies, duration of longitudinal follow-up and utilized liquid biopsy techniques. Importantly, detection of molecular recurrence preceded radiologic recurrence by median lead times ranging from 2.3 months to more than 1 year^63^, underscoring the potential utility of longitudinal strategy as an “early warning approach”. A notable exception to these promising results may be patients with brain-only recurrence, as shown in the study by Zhang et al. (longitudinal analysis NPV: 20%)^48^ and reported in other solid tumors as well^64^. Nevertheless, the clinical utility of early interception in disease progression using liquid biopsy requires further investigation within large RCTs.

### What is the clinical utility of MRD in guiding adjuvant/consolidation treatment strategies in NSCLC?

(Neo)adjuvant or perioperative chemoimmunotherapy aims at the eradication of potential micrometastases and represents the current standard of care for resectable NSCLC^17,65^. The same holds true for consolidation immunotherapy for patients with locally advanced, unresectable disease that achieve complete response after definitive chemoradiotherapy^66^. Technological advances in MRD detection, along with the recognition of its strong prognostic potential, have given rise to the concept of MRD-guided adjuvant/consolidation therapy in solid tumors, including NSCLC^67^.

ctDNA analysis could be used to de-escalate therapy in resectable NSCLC in order to avoid overtreatment and unnecessary toxicity of intensive multimodality therapies for patients at low risk for disease progression. This concept becomes even more relevant following the recent concern of the FDA Oncologic Drugs Advisory Committee regarding potential overtreatment with perioperative regimens in resectable disease, and the recommendation for a redesign of perioperative immunotherapy trials that will allow for the relative contributions of the neoadjuvant and adjuvant phases to be more clearly defined^68^.

In a recent meta-analysis of 6 NSCLC studies (2 of which included patients with locally advanced, unresectable disease^69,70^), using predictive values analysis, it was concluded that ctDNA(-) patients did not derive RFS benefit from adjuvant/consolidation therapy (HR: 1.51, 95% CI: 0.81–2.79, *p* = 0.19)^41^. A notable exception was adjuvant Atezolizumab that increased DFS (*vs*. placebo) in patients with stage IB-IIIA NSCLC and PD-L1 tumor cell (TC) score ≥1% (but not < 1%), regardless of the ctDNA status [ctDNA(-): HR for DFS: 0.57, 95% CI: 0.36–0.9; ctDNA(+): HR for DFS: 0.54, 95% CI: 0.31–0.93]^49^.

Successful MRD-guided treatment de-escalation was recently demonstrated in a proof-of-concept prospective, non-randomized controlled trial (LOCAL) that included patients with metastatic or locally advanced inoperable, oncogene-driven NSCLC. All enrolled subjects had no radiologic evidence of disease after local consolidative and TKI induction therapies, and were serially evaluated for MRD (along with CEA levels). Patients with longitudinally undetectable ctDNA received a drug holiday, displaying excellent survival outcomes (median PFS not reached; no deaths recorded)^71^. In the same study, treatment was successfully re-escalated in the subgroup of patients who converted to MRD(+) status (objective response rate after treatment reinitiation: 96%). In early NSCLC, de-escalation of adjuvant therapy has been evaluated in patients using the perioperative detection of clusters of CTCs. In the CTC(–) patient group, administration of adjuvant chemotherapy did not improve 2-year RFS (94.9% *vs*. 90.9%), whereas CTC(+) patients derived considerable benefit (71.8% *vs*. 36.3%, *p* < 0.1)^72^.

The potential clinical use of ctDNA analysis in guiding safe treatment de-escalation of adjuvant/consolidation therapy presupposes that liquid biopsy can reliably discriminate MRD(-), “cured” patients without micrometastatic disease from those who are still at risk of recurrence. In the study by Zhang et al., NSCLC patients that received radical treatment and retained longitudinally negative MRD measurements over 18 months were defined as potentially cured based on the remarkably high NPV of longitudinal analysis for disease recurrence (>95%)^48^. In the same study, the peak risk of detectable MRD was approximately 12–18 months after landmark detection which significantly constrains its use, since in the clinic, decision-making about additional therapy (i.e. adjuvant therapy) needs to be undertaken no later than 4–8 weeks after surgery. On the other hand, if a landmark strategy is opted for, the optimal timing for MRD evaluation remains uncertain. It has been suggested that a longitudinal strategy consisting of 2-3 MRD evaluations within 1 month after curative-intent therapy may represent the optimal risk stratification approach before deciding upon (de)-escalation of further treatment modalities^73,74^. Considering the existence of low- or non-shedding tumors, ctDNA tests before surgery may be used to assess tumor DNA shedding potential for each individual. Finally, adopting a treatment de-escalation strategy in routine care requires confirmation of non-inferiority from adequately powered randomized clinical trials, whereas, consensus has to be reached for the appropriate non-inferiority margin. A randomized pilot study of ctDNA-guided Osimertinib maintenance *vs*. observation alone in resected *EGFR*-mutated disease is currently recruiting patients (*ECTOP-1022*, Table 2).

**Table 2:** List of active clinical trials evaluating MRD-guided adaptive management of non-metastatic NSCLC

Disease Stage	Phase	Measurement strategy (MRD)^#^	Molecular background	Accrual (est.)	Study arms & interventions	Primary endpoint(s)	Status	Trial Id
I	n/a	Landmark	ns	342	A (MRD+): Treatment B (MRD-): Observation	3-yr DFS	Not yet recruiting	NCT06709274 (MOTION-NSCLC)
IA-IIA	II	Landmark	*EGFR*-mutant	32	A (MRD+): Osimertinib or Observation B (MRD-): Observation	PFS	Not yet recruiting	(No NCT no.) PMID: 39659920
IB-IIIA	Obs.	Longitudinal	ns	180	(MRD-): Observation (single-arm)	2-yr DFS	Recruiting	NCT05457049 (CTONG 2201)
I-III	II	Landmark	Non-mutant	80	A (MRD+): Durvalumab (2 C) +/− maintenance Durvalumab B (MRD-): Observation	Decrease in ctDNA	Recruiting	NCT04585477 (ADAPT-E)
I-III	II	Landmark	ns	66	A (MRD+): Chemotherapy B (MRD+): Observation	2-yr RFS	Recruiting	NCT04966663 (ctDNA Lung RCT)
IB-IIIB	II	Longitudinal	*EGFR*-mutant	180	A (MRD+): Icotinib (on/off) B (MRD-): Observation	3-yr DFS	Recruiting	NCT05536505
IB-IIIA	II	Landmark	Non-mutant	80	A (MRD+)^*^: Toripalimab B (MRD-)^*^: Observation	2-yr DFS	Recruiting	NCT06426511 (CONTINUE)
II-IIIA	III	Landmark	*EGFR*-mutant	226	A (MRD+): Osimertinib B (MRD+): Observation	3-yr DFS	Not yet recruiting	NCT06323148 (ECTOP-1022)
IB-IIA	Obs.	Longitudinal	ns	150	A (MRD+): Chemotherapy or Observation B (MRD-): Chemotherapy or Observation	3-yr DFS	Recruiting	NCT05167604
III	III	Longitudinal	*EGFR*-mutant	192	A: Continuous Almonertinib treatment B: MRD-guided Almonertinib treatment (on/off)	18-mo EFS, 8-wk ORR	Recruiting	NCT04841811 (APPROACH)
III	III	Landmark	ns	48	A (MRD+): Chemotherapy + Durvalumab + Tremelimumab (4 C) +/− maintenance Durvalumab B (MRD-): Durvalumab	Change in ctDNA after ChT	Recruiting	NCT04585490

All studies utilized ctDNA to measure MRD.

Source: ClinicalTrials.gov.

*C* cycle, *ChT* chemotherapy, *ctDNA* circulating tumor DNA, *DFS* disease-free survival, *EFS* event-free survival, *EGFR* Epidermal Growth Factor Receptor, *mo* month, *MRD* minimal residual disease, *n/a* not applicable, *no*. number, *ns* not specified, *Obs*. observational, *ORR* objective response rate, *PFS* progression-free survival, *RFS* recurrence-free survival, *wk* week, *yr* year.

^#^Refers to the number of MRD measurements before the initial treatment allocation.

*Refers to the MRD status after completion of 4 cycles of Toripalimab + Chemotherapy.

Inversely, MRD-guided treatment intensification, a challenging concept not limited to NSCLC, could be applied in high-risk patients with baseline ctDNA detection and/or lack of ctDNA clearance after curative treatment. Importantly, treatment intensification could also be relevant for the poor-risk patients with stage I disease exhibiting preoperative ctDNA release^52^. Moreover, in the case of immunotherapy, there is evidence that even ctDNA(–) patients may derive survival benefit, as already shown for Atezolizumab in the adjuvant setting^49^.

The recent paradigm of the PADA-1 trial in metastatic, hormone receptor-positive, HER2-negative breast cancer (on-treatment switch from letrozole to fulvestrant)^75^ suggested the feasibility and clinical utility of liquid biopsy-guided escalation of treatment. In NSCLC, there is relative paucity of data to support or disprove the value of MRD-based treatment escalation strategies. Moreover, it is unclear whether treating patients based on MRD positivity alone can affect the natural history of the disease. The first attempt to answer these questions was the phase III MERMAID-2 trial, which aimed to assess the effect on DFS from addition of Durvalumab (escalation arm) *vs*. placebo (control arm) in patients with resected stage II-III NSCLC who tested MRD(+) after curative-intent therapy^76^. The study was prematurely terminated after the introduction of immunotherapy to the standard perioperative care of early-stage NSCLC patients, rendering the control arm outdated. Nevertheless, the phase II APPLE study provided promising results in patients with advanced, *EGFR*-driven NSCLC. In this study, serial monitoring of ctDNA during Gefinitib treatment identified 17% of patients with molecular progression (emergence of the T790M mutation) before RECIST progression, leading to an earlier switch to Osimertinib with a clinically meaningful 18-month PFS rate of 67%^77^.

In view of the existing evidence supporting the potential clinical utility of ctDNA-based individualization of treatment, novel large interventional trials are being conducted in early-stage and locally advanced, resectable NSCLC, in order to further elucidate the role of ctDNA in guiding (neo)adjuvant treatment decisions, minimizing both clinical and financial toxicities [Table 2].

## Conclusions and future directions

Liquid biopsy has emerged as a versatile tool in NSCLC, which, besides its diagnostic utility, allows monitoring of disease activity at the molecular level through detection of MRD, compensating for the often limited sensitivity of imaging techniques^78^. Specifically, ctDNA represents a valid tool for predicting the risk of relapse in resected NSCLC, with multiple studies demonstrating a strong association between ctDNA positivity and decreased RFS and OS. Importantly, sensitivity in predicting relapse may improve when post-treatment MRD evaluation is conducted across sequential follow-up time points (longitudinal surveillance) rather than restricted to a single assessment. This evidence positions MRD assessment as a promising new tool for risk stratification and individualized management of patients with NSCLC receiving curative-intent treatment that could potentially enable early identification and treatment intensification for patients at high risk of relapse, while potentially reducing overtreatment for those who may have already achieved cure. However, despite significant advancements, the implementation of ctDNA in early-stage NSCLC remains limited, requiring further refinement of technical and clinical protocols.

Improving the sensitivity of MRD detection remains a key challenge, considering the high false-negative rates (15–61%) and the substantial costs associated with achieving sufficient test sensitivity. Detecting very low MAFs (as low as 0.01%) is critical but currently feasible in only about 50% of patients with recurrent disease^79^. Low-shedding situations, such as stage I NSCLC, nodal-^80^ or brain-only recurrent disease, present additional challenges. Although novel, ultrasensitive technologies like PhasED-Seq^81^ have shown promise, extensive validation across various clinical settings is required. Strategies, such as increasing ctDNA input^40^ and leveraging longitudinal surveillance are under investigation to enhance recurrence detection. Apart from its biological rarity, operational barriers such as assay standardization, variable sensitivity thresholds, and limited regulatory approvals for MRD-specific applications further hinder clinical applicability. While FDA-approved platforms like Guardant360 CDx™ and FoundationOne Liquid CDx™ have been validated for therapy selection when tissue is not available or at disease progression, their data supporting MRD use remain insufficient.

Large prospective randomized trials are needed to establish the clinical utility of ctDNA assessments in early NSCLC. Proposed trial methodologies vary, i.e. some may randomize ctDNA(+) patients between standard-of-care treatment and intensified regimens, while others may explore treatment de-escalation in ctDNA(-) patients^67^. It is conceivable that MRD-guided treatment de-escalation strategies need to demonstrate non-inferiority in terms of survival before entering routine clinical practice. Currently, most studies have so far focused on the refinement of adjuvant treatment strategies; however, one could foresee the potential utility of liquid biopsy for the decision-making process in the neoadjuvant setting as well (e.g., decision for surgery *vs*. treatment prolongation based on ctDNA clearance). The previously described discordance between ctDNA clearance and pCR will probably require integrated biomarker strategies that combine ctDNA dynamics with histopathologic and imaging assessments. An important consideration that is applicable to both adjuvant and neoadjuvant settings is the establishment of the optimal time point for MRD assessment and the selection of landmark or longitudinal approaches in order to refine risk stratification and ensure clinically meaningful outcomes. Finally, the potential extension of MRD-guided escalation strategies to early-stage IA patients, typically excluded from adjuvant therapies, represents another unexplored area of interest, requiring highly sensitive detection methods.

A critical open question is whether the early detection of molecular recurrence via ctDNA translates into improved patient outcomes. Although several exploratory analyses have correlated ctDNA clearance or conversion with treatment efficacy, the role of ctDNA as a surrogate marker for survival requires validation within well-designed RCTs with representative patient populations^82^. Perhaps, integration of ctDNA monitoring with intensified imaging, such as early CT or PET/CT scans for patients with ctDNA positivity, could potentially improve outcomes. Such an approach has already demonstrated promising results in small cohorts of resected NSCLC patients^83^, underscoring the potential of combining molecular and radiologic surveillance.

Overall, this review highlights the transformative potential of MRD detection to guide decision-making in non-metastatic NSCLC. However, most evidence to date stem from retrospective or exploratory analyses, rather than prospective, randomized trials. Future trials must address these gaps, focusing on feasibility, clinical utility and costs of ctDNA analyses in different scenarios. Lessons from other malignancies, such as stage II CRC^84^, where ctDNA has shown great promise, may further inform the pathway towards establishing the role of MRD detection for precision oncology in NSCLC.

## Data Availability

No datasets were generated or analysed during the current study.
